# Heparan Sulfate Biosynthetic System Is Inhibited in Human Glioma Due to *EXT1/2* and *HS6ST1/2* Down-Regulation

**DOI:** 10.3390/ijms18112301

**Published:** 2017-11-01

**Authors:** Victor S. Ushakov, Alexandra Y. Tsidulko, Gabin de La Bourdonnaye, Galina M. Kazanskaya, Alexander M. Volkov, Roman S. Kiselev, Vyacheslav V. Kobozev, Diana V. Kostromskaya, Alexey S. Gaytan, Alexei L. Krivoshapkin, Svetlana V. Aidagulova, Elvira V. Grigorieva

**Affiliations:** 1 Institute of Molecular Biology and Biophysics, Novosibirsk 630117, Russia; v.ushakov.nsu@gmail.com (V.S.U.); sephellone@gmail.com (A.Y.T.); g_kazanskaya@meshalkin.ru (G.M.K.); 2 Novosibirsk State University, Novosibirsk 630090, Russia; gabindelab@gmail.com; 3 National Institute of Applied Sciences, 31400 Toulouse, France; 4 Meshalkin National Medical Research Centre, 630055 Novosibirsk, Russia; a_volkov@meshalkin.ru (A.M.V.); rayjelly@gmail.com (R.S.K.); v_kobozev@meshalkin.ru (V.V.K.); d_kostromskaya@meshalkin.ru (D.V.K.); alkr01@yandex.ru (A.L.K.); 5 Novosibirsk State Medical University, 630090 Novosibirsk, Russia; a_sv@ngs.ru; 6 European Medical Centre, 129110 Moscow, Russia; lanceter@mail.ru

**Keywords:** heparan sulfate, biosynthesis, sulfotransferase, heparanase, glioma, invasion, extracellular matrix, tumour microenvironment

## Abstract

Heparan sulfate (HS) is an important component of the extracellular matrix and cell surface, which plays a key role in cell–cell and cell–matrix interactions. Functional activity of HS directly depends on its structure, which determined by a complex system of HS biosynthetic enzymes. During malignant transformation, the system can undergo significant changes, but for glioma, HS biosynthesis has not been studied in detail. In this study, we performed a comparative analysis of the HS biosynthetic system in human gliomas of different grades. RT-PCR analysis showed that the overall transcriptional activity of the main HS biosynthesis-involved genes (*EXT1*, *EXT2*, *NDST1*, *NDST2*, *GLCE*, *HS2ST1*, *HS3ST1*, *HS3ST2*, *HS6ST1*, *HS6ST2*, *SULF1*, *SULF2*, *HPSE*) was decreased by 1.5–2-fold in Grade II-III glioma (*p* < 0.01) and by 3-fold in Grade IV glioma (glioblastoma multiforme, GBM) (*p* < 0.05), as compared with the para-tumourous tissue. The inhibition was mainly due to the elongation (a decrease in *EXT1/2* expression by 3–4-fold) and 6-*O*-sulfation steps (a decrease in *6OST1/2* expression by 2–5-fold) of the HS biosynthesis. Heparanase (*HPSE*) expression was identified in 50% of GBM tumours by immunostaining, and was characterised by a high intratumoural heterogeneity of the presence of the HPSE protein. The detected disorganisation of the HS biosynthetic system in gliomas might be a potential molecular mechanism for the changes of HS structure and content in tumour microenvironments, contributing to the invasion of glioma cells and the development of the disease.

## 1. Introduction

Malignant brain tumours still remain one of the most dangerous cancer types. Their high aggressiveness is primarily determined by the active invasion of the growing tumour into the surrounding normal brain tissue; this depends on both the invasive potential of the tumour cells and the structure of the extracellular matrix (ECM), comprising about 20% of the normal brain tissue [1,2]. Brain ECM consists mainly of complex protein-polysaccharide molecules of proteoglycans (PGs), the structure and composition of which play critical roles in development and normal function of the central nervous system [3,4]. Chondroitin sulfate (CS) and hyaluronic acid (HA) are two major glycosaminoglycans (GAGs) present in the brain ECM [5,6], and for a long time, neuroscience research has been focused mainly on the function of CS and HA in brain physiology and pathology. However, emerging evidence also suggests an important role for the other PGs/GAGs in regulating CNS function in normal and pathological conditions [4].

One of the key components of ECM and cell surface of almost all mammalian tissues is heparan sulfate (HS), which comprises long linear polysaccharide chains with a high negative charge and extremely heterogeneous structure [7,8]. Heparan sulfate proteoglycans (HSPGs) carrying HS polysaccharide chains form the structure of ECM, participate in the cell-cell and cell-matrix interactions, and ensure migration activity of the cells and their binding to various growth factors and cytokines [9,10,11]. In the nervous system, HS interacts with a variety of physiologically important macromolecules, and plays a significant role in brain development and normal functioning, while undergoing changes during malignant transformation [12,13,14]. Different glioblastoma primary cell cultures have been shown to possess a high heterogeneity of the HS structure and composition, which has been associated with the adhesive properties and invasive activity of these cells [15]. However, molecular mechanisms of deregulation of HS in gliomas remain poorly understood, and appear to be determined by the balance of the processes of biosynthesis of HS and its extracellular degradation [16].

Since the biosynthesis of HS is performed in a non-template manner by a complex system of biosynthetic enzymes, disturbances in its work may be the most likely cause of changes in the structure and/or composition of HS in tumours [17,18]. The transcriptional activity of this system is tissue-specific [19], and it is under control of complex epigenetic mechanisms [20]. There have been practically no comprehensive studies of the HS biosynthetic system in gliomas, and the only known analysis of the expression pattern of HS biosynthesis-related genes in glioblastoma was carried out by Wade et al., 2013, using data from the Cancer Genome Atlas (available online: http://cancergenome.nih.gov/) [21]. The expression of HS biosynthetic enzymes, including HS6ST1–3, has been shown to be predominantly down-regulated in glioblastoma, with the striking exceptions of HS3ST3a1 and the extracellular HS modifying enzyme HPSE [22].

As to the individual enzymes of HSbiosynthesis, there is also only limited data on the expression of enzymes responsible for extracellular modifications of HS-sulfatases and heparanase. In a model of experimental animals, the correlation of *SULF2* expression with PDGFRα phosphorylation and decreased MAPK signaling has been shown, whereas the knockout of *Sulf2*(−/−) resulted in an inhibition of tumour growth in experimental animals in vivo [23]. Increased expression of sulfatase in the neurospheres of primary cultures of human and mouse gliomas was associated with a low content of trisulfated disaccharides of HS in the system in vitro [15], while increased expression of *SULF1* and *SULF2* in brain cancer was associated with progression and prognosis of the disease in vivo [24]. At the same time, expression of *SULF1* and *SULF2* demonstrates subtype-specific differences, and can be both significantly reduced in classical GBM and significantly elevated in proneural GBM, suggesting different functions in GBM subgroups [22].

Heparanase is the most studied enzyme among those responsible for the HS metabolism. It is one of the key regulators of cancer progression, tumour angiogenesis and metastasis [25,26,27]. Nevertheless, the data on heparanase in gliomas are not very abundant, and are contradictory. The activation of heparanase expression in human U251n glioma cell lines in vitro has been shown to significantly increase cell invasion, proliferation, anchorage-independent colony formation and chemotactic migration of these cells [28]. In human U87 glioblastoma cell lines, an increase in heparanase expression also enhances the invasive potential of cells, but reduces their proliferative activity, facilitates cell spreading and monolayer formation. At the same time, the growth of experimental tumoursin vivo depends on the level of ectopic expression of heparanase; a moderate level of expression enhanced tumour growth, while a high level suppressed it [29]. According to Ueno et al., heparanase is expressed in the U87MG and U251MG cell lines in vitro, but is virtually undetectable in the grown experimental tumoursin vivo, and it does not appear to contribute to the invasivity of glioma cells [30]. In the experimental models of HPSE transgenic and knockout mice, the activation of heparanase changes the microenvironment of tumour and positively correlates with its development, while the suppression of heparanase reduces the amount of tumour cells. A high level of heparanase expression has also been shown in human gliomas, as compared to normal brain tissue, which correlates with a shorter survival of patients with a high-grade glioma [31]. Hong et al. also observed an elevated level of heparanase expression in gliomas in vivo, which various types of cells contribute to—endothelial cells, neutrophils and, to some extent glioma cells, the expression of heparanase in which is associated with an elevated Ki67 index [32].

In this work, we aimed to investigate the HS biosynthetic system in gliomas of different grades (astrocytoma, anaplastic astrocytoma of Grades II-III, glioblastoma multiforme (GBM) of Grade IV) and evaluate its role in the disease development.

## 2. Results

### 2.1. Transcriptional Activity of HS(Heparan sulfate)Biosynthetic System Is Inhibited in Glioma

**Figure 1:**
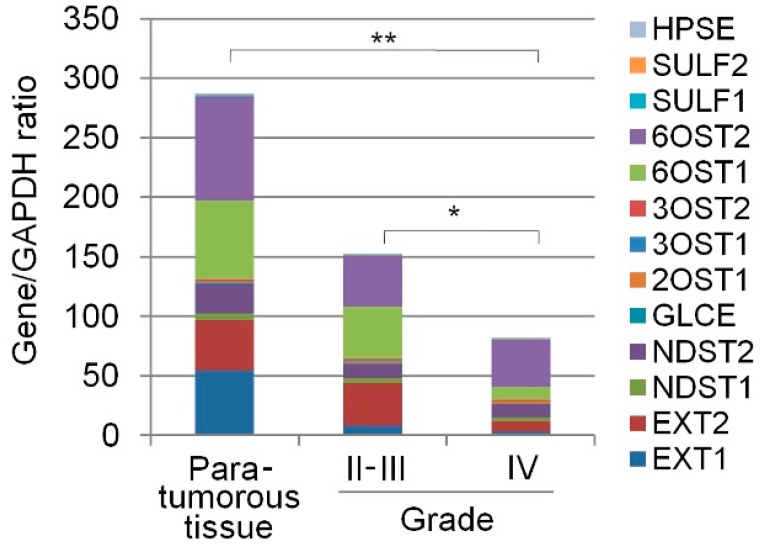
Transcriptional activity of the HS biosynthetic system in gliomas of different grades. The intensity of the amplified DNA fragments of HS biosynthesis-related genes was normalised to that of *GAPDH*. The stacked columns reflect the contribution of each gene to the total expression level. Control—para-tumourous brain tissuefrom the Grade II glioma patients. The stacked columns are based on the mean expression levels from triplicate experiments (Origin 8.5; OriginLab Corporation, Northampton, USA); * *p* < 0.01; ** *p* < 0.05.

Expression of the main enzymes responsible for heparan sulfate biosynthesis and post-synthetic modification (EXT1, EXT2, NDST1, NDST2, GLCE, 2OST1/HS2ST1, 3OST1/HS3ST1, 3OST2/HS3ST2, 6OST1/HS6ST1, 6OST2/HS6ST2, SULF1, SULF2, HPSE) was determined in human gliomas of various degrees of malignancy (astrocytoma, Grade II, *n* = 9; anaplastic astrocytoma, Grades III, *n* = 5; glioblastoma, Grade IV, *n* = 28) by Real-Time RT-PCR (Figure 1).

Despite the high variability of individual tumours in the expression levels of heparan sulfate biosynthesis-related genes, the overall transcriptional activity of the HS biosynthetic system was decreased by 1.5–2-fold (*p* < 0.01) in gliomas of Grade II-III and by 1.5–2-fold (*p* < 0.01) in GBM compared with the para-tumourous brain tissue from the Grade II glioma patients (*n* = 3) (Figure 1 and Table 1).

### 2.2. Elongation and 6-O-Sulfation Steps Are Main Contributors to HS Biosynthetic System Inhibition

**Figure 2:**
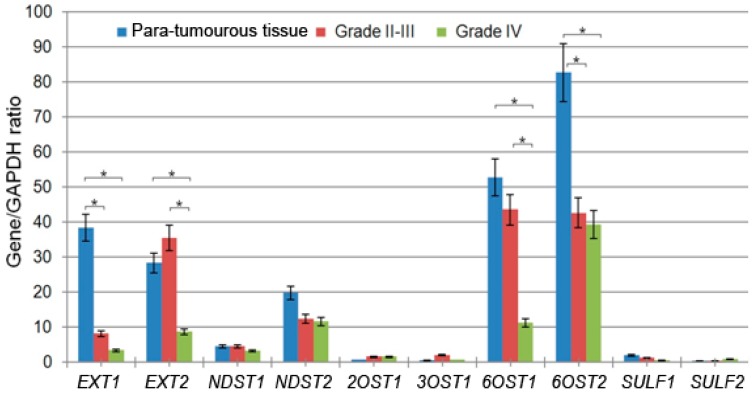
Expression of HS biosynthesis-related genes in human gliomas of different grades. The intensity of the amplified DNA fragments was normalised to that of *GAPDH*. The graphs show the mean expression levels from triplicate experiments ± SD (Origin 8.5; OriginLab Corporation, Northampton, USA); * *p* < 0.01.

The most significant contribution to the overall inhibition of the system was due to down-regulation of the expression of genes responsible for elongation of the HS chains (exostosin glycosyltransferases *EXT1* and *EXT2*) and its sulfation at the 6-*O*-position (6-*O*-sulfotransferases *6OST1* and *6OST2*) (Figure 2). These genes were the most expressed ones in the para-tumourous brain tissues and their expression levels were significantly decreased in gliomas, depending on the tumour grade. In Grade II-III tumours, there was a 4-fold decrease in the EXT1 expression level, though the EXT2 expression was maintained, whereas in GBM tissues (Grade IV) the EXT2 expression level was decreased by 3–4-fold as well (*p* < 0.05). Similar results were obtained for 6-*O*-sulfotransferases–the 6OST2 expression was decreased by 2-fold in Grade II-III gliomas with the 6OST1 expression still maintained, whereas in GBM the 6OST1 expression was decreased by 4–5-fold as well (Figure 2 and Table 1).

### 2.3. Heparanase Expression in Glioblastoma Possesses Both Inter- and Intratumour Heterogeneity

**Figure 3:**
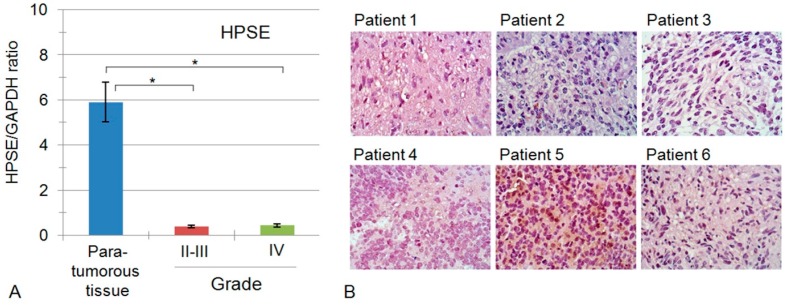
*HPSE* expression in the human brain tumours. (**A**) HPSE mRNA expression levels in tumour samples (real-time RT-PCR). The intensity of the amplified DNA fragments was normalised to that of *GAPDH*. Bars represent the mean from triplicate experiments ± SD (Origin 8.5; OriginLab Corporation, Northampton, USA); * *p* < 0.01; (**B**) Expression and distribution of HPSE protein molecule in glioblastoma tissues. Immunostaining of GBM tumours using anti-HPSE antibodies; counterstaining with Hematoxylin; 1–6 patients; magnification ×400.

The transcription activity of the other HS biosynthesis-related genes under the study was low and did not differ significantly in gliomas and para-tumourous brain tissues, except for the heparanase gene (*HPSE*). According to the obtained data, relatively low *HPSE* expression level in para-tumourous brain tissue was further significantly decreased in gliomas (*p* < 0.01), and the effect was not associated with the tumour grade (Figure 3A). The immunohistochemical analysis (IHC) was performed for glioblastoma (Grade IV) tissues only, and no staining for the HPSE protein molecule was shown for a near a half of the analysed GBM tumours, while the other 50% of the GBM tumours demonstrated positive signal for HPSE protein (Figure 3).

**Figure 4:**
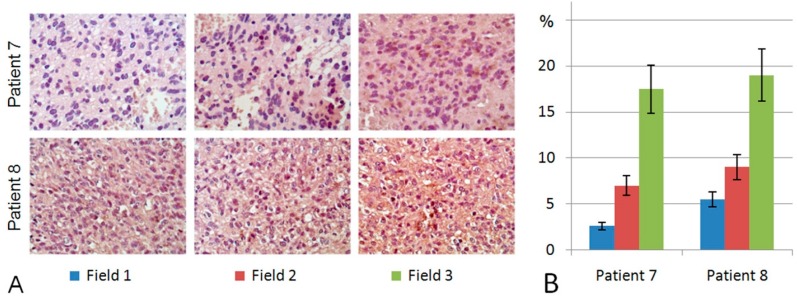
Intratumour heterogeneity of *HPSE* expression in glioblastoma. (**A**) Immunostaining of GBM tumours using anti-HPSE antibodies. Patients 7 and 8; Fields 1,2,3-observation fields of the histological preparation; counterstaining with Hematoxylin; magnification ×400; (**B**) Semi-quantitative analysis of anti-HPSE staining intensities in different observation fields. Bars represent the mean from triplicate experiments ± SD (Origin 8.5; OriginLab Corporation, Northampton, USA); 7, 8-patients.

Interestingly, in addition to the heterogeneity of individual glioblastoma tumours for the presence/absence of HPSE expression, a high intratumoural heterogeneity of anti-HPSE staining was revealed in HPSE-positive ones (Figure 4).

The intensities of anti-HPSE staining varied in different areas of the same GBM tumour by up to 4–5-fold, demonstrating a high intratumour heterogeneity of HPSE expression in glioblastoma (Grade IV) tissues, which contributes to the high overall heterogeneity of glioblastoma cells within individual tumours (Figure 4). In many cases, the presence of HPSE was more typical of relapsing tumours than the corresponding GBM clinical samples obtained during the first surgery for the same patient; however, this observation needs to be confirmed on a larger number of tumour samples.

Taken together, the obtained data demonstrate a significant suppression of the transcriptional activity of the HS biosynthetic system in human gliomas, which depends on the extent of tumour malignancy and is based mainly on the inhibition of elongation and 6-*O*-sulfation steps of HS biosynthesis. The heterogeneous expression of heparanase in individual glioblastoma tumours might contribute to the HS content in these tissues and structure of the tumour microenvironment.

## 3. Discussion

Due to insufficient knowledge of the HS biosynthetic system in gliomas, the obtained results can be compared only with the data on the analysis of the expression pattern of HS biosynthetic enzymes in glioblastoma from the Cancer Genome Atlas [22]. Our results on the decreased overall transcriptional activity of the HS biosynthetic system in glioblastoma (Figure 1) are in good agreement with the TCGA-based analysis. They also correlate with and supplement the data on the tissue specificity of changes in the transcriptional activity of the HS biosynthetic system in various cancer types; a significant suppression of this system has been shown in human prostate tumours [33] and Burkitt’s lymphoma cells [34], although its activity was not changed in breast and colon tumours [19,35]. At the same time, there are some contrasts between our results and the earlier published data for individual genes. A significant decrease in the *EXT1* and *EXT2* expression levels in gliomas was demonstrated here (Figure 2), but according to Wade et al. [22], expression of these genes is increased in glioblastoma; low expression levels of sulfatases (*SULF1*, *SULF2*) in the para-tumourous brain tissue were not changed in gliomas of different grades, which contradicts the limited published data on increased expression of *SULF1* and *SULF2* in brain cancer [23,24]. Such discrepancies may result from methodological differences in measuring gene expression, or detection of various alternatively spliced mRNA isoforms.

Of particular interest are the presented data on down-regulation of the transcriptional activity of the genes involved in the 6-*O*-sulfation of HS (*6OST1*/*HS6ST1*, *6OST2*/*HS6ST2*), since the sulfation contributes significantly to the structural diversity of the HS chains and plays a crucial role in its binding to specific ligands and maintenance of cell-cell and cell-matrix interactions in normal and pathological conditions [36,37]. The results are in good agreement with the Cancer Genome Atlas data [22], and have similarities to the data by Yamada et al. about a decrease in the 6-*O*-sulfation of HS in ageing brain tissue [38]. They reveal a similar trend on *6OST1*/*6OST2* down-regulation in malignant brain tumours, and suppression of 6-*O*-sulfation in the aged brain tissue, which supports an important role of 6-*O*-sulfation in normal brain physiology. The inhibition of 6-*O*-sulfation in gliomas, along with the demonstrated absence of significant expression of 2-*O*- and 3-*O*-sulfotransferases, suggests a potential decrease of the sulfation of HS chains in glial tumours and attenuation of their functional activity in the tumour microenvironment.

Heparanase (HPSE), an enzyme responsible for the cleavage of polysaccharide chains of HS in the extracellular matrix and on the cell surface, affects the HS content in gliomas and tumour microenvironments, as well [39]. Although a decreased HPSE expression was detected at the mRNA level, immunohistochemical analysis demonstrated the presence of HPSE protein molecules in 50% of the studied glioblastoma tumours (Figure 3 and Figure 4). The difference in HPSE expression in individual GBM tumours stratifies them into two sub-groups (HPSE-positive and HPSE-negative) with potentially different clinical characteristics, which might correspond to Kundu’s data on the correlation of a high level of heparanase expression with low survival of patients with glioblastoma [31], supplementing them with new data on the high intratumoural heterogeneity of HPSE distribution in glioblastoma tissues.

Another important aspect of the study is related to the extracellular and/or intracellular localisation of HPSE and its association with certain cell types. It is known that different cell types contribute to the increased HPSE expression level in gliomas in vivo (endothelial cells, neutrophils and to some extent glioma cells, the expression of heparanase in which is associated with an elevated Ki67 index) [32]. In our study, a predominantly extracellular localisation of anti-HPSE staining was observed, although some glioma cells expressed HPSE as well, supporting the data on the high HPSE expression in glioblastoma cell lines U87 and U251 in vitro [28,29,30].

In summary, heparan sulfate biosynthetic system and heparanase contributes to a complex and heterogeneous nature of glioblastoma tumours, and their study requires consideration both the expression levels of HS biosynthesis-related genes and intratumour localisation of the corresponding protein molecules in tumour cells, cells of tumour microenvironment or extracellular matrix. Therefore, a separate analysis of the appearance/disappearance of HPSE expression in glioma cells or cells of the tumour microenvironment is warranted.

## 4. Materials and Methods

### 4.1. Patients and Tissue Samples

All tissue samples were obtained from brain tumours during radical surgery at the Neurosurgical Unit of Meshalkin National Medical Research Centre, Novosibirsk, Russia. Tissues were collected in RNAlater (Thermo Fisher Scientific, Waltham, MA, USA) and stored at −20 °C or fixed in buffered 4% formalin solution and embedded in paraffin. H&E-stained sections were prepared to define representative tumour regions and GBM diagnosis was confirmed according to the World Health Organization (WHO) classification by qualified pathologists. For RT-PCR, regions were manually dissected from the tissue samples to provide a consistent tumour cell content of more than 70% for analysis. In total, 42 patients were used in the study (14 cases of astrocytoma and anaplastic astrocytoma (WHO Grade II–III), 28 cases of glioblastoma (WHO Grade IV). Para-tumourous tissues were obtained from the peritumoural area of Grade II glioma. All the patients received only adjuvant therapy (temozolomide, 5 days/month repeating courses, in combination with radiotherapy, 30 days, 2 Gy/day, totally 60 Gy) to prevent residual glioma cell dissemination and disease relapse.

Informed consent was obtained from all individual participants included in the study. The study protocol was approved by the Local Ethics Committee in accordance with the Helsinki Declaration of 1975. Clinical information is presented in Table 2.

### 4.2. RT-PCR Analysis

Total RNA was extracted using TRIzol Reagent (ThermoFisher Scientific, Waltham, MA, USA) according to the manufacturer’s instructions. The integrity and quality of the isolated RNA was determined by agarose gel electrophoresis, the RNA concentration was determined using a Qubit fluorimeter and Quant-iT RNA Assay Kit (ThermoFisher Scientific) according to the manufacturer’s instructions.

cDNA was synthesised from 1 µg of total RNA using a Maxima First Strand cDNA Synthesis kit (Fermentas, Hanover, MD, USA) and 1/10th of the product was subjected to PCR analysis.

SYBRGreen-based real-time PCR was performed using the CFX96 Touch Real-Time PCR Detection System (Bio-Rad, Hercules, CA, USA) under the following conditions: 95 °C for 2 min, followed by 40 cycles at 95 °C for 20 s, 59 °C for 15 s and 72 °C for 50 s. The total reaction volume was 25 μL. Glyceraldehyde 3-phosphate dehydrogenase (*GAPDH*) was used as the housekeeping gene. The PCR primers are described in Table 3.

### 4.3. Immunohistochemical Analysis

For immunohistochemistry, 3- to 3.5-μm sections of formalin-fixed paraffin-embedded tissue sections were treated with sodium citrate buffer (10 mM sodium citrate, 0.05% Tween-20) at 95–98 °C for 20 min for antigen unmasking. The anti-HPSE rabbit monoclonal antibody (Abcam; 1:100) was used for immunostaining. Staining patterns were visualised with Histostain-Plus 3rd Gen IHC Detection Kit (ThermoFisher Scientific). The sections were counterstained with Hematoxylin and observed by light microscopy using AxioScopeA1 microscope (Zeiss, Oberkochen, Germany). Quantitative analysis was perform with ZENblue program (Zeiss)

### 4.4. Statistical Analysis

Statistical analyses were performed using ORIGIN 8.5 software (OriginLab Corporation, Northampton, MA, USA); a value of *p* < 0.05 was considered to indicate a statistically significant difference. Data are expressed as the means ± SD.

## 5. Conclusions

The revealed complex inhibition of the transcriptional activity of HS biosynthetic system in human glioma tumours might result in the deterioration of both the structure (due to 6-*O*-sulfatase down-regulation) and content (due to HS elongation-related gene down-regulation) of heparan sulfate in glioblastoma tissues. Heterogeneous expression of heparanase in GBM tumours suggests additional regulation of HS content in the glioma tissues at the extracellular level. Taken together, these changes might contribute to the transformation of the normal brain ECM into the tumour microenvironment and formation of a tumourigenic niche for glioma development and disease progression.

## Figures and Tables

**Table 1:** Expression levels of HS biosynthesis-involved genes in glioma.

Gene ID	Primary Source	Gene Name	Description	Para-Tumourous Tissue	Grade II-III	Grade IV
2131	HGNC:3512	*EXT1*	Exostosin Glycosyltransferase 1	54.9 ± 58	8.2 ± 8.38	3.4 ± 2.1
2132	HGNC:3513	*EXT2*	Exostosin Glycosyltransferase 2	41.5 ± 54	35.5 ± 54	8.7 ± 5.7
3340	HGNC:7680	*NDST1*	*N*-deacetylase and *N*-sulfotransferase 1	6.2 ± 2	4.5 ± 7	3.2 ± 6
8509	HGNC:7681	*NDST2*	*N*-deacetylase and *N*-sulfotransferase 2	25.5 ± 4	12.4 ± 8	11.6 ± 14
26035	HGNC:17855	*GLCE*	Glucuronic Acid Epimerase	0.001 ± 0.001	0.005 ± 0.008	0.002 ± 0.001
9653	HGNC:5193	*2OST1*/*HS2ST1*	Heparan Sulfate 2-*O*-Sulfotransferase 1	1.12 ± 1.4	1.6 ± 1.33	1.58 ± 1.1
9957	HGNC:5194	*3OST1*/*HS3ST1*	Heparan Sulfate-Glucosamine 3-*O*-Sulfotransferase 1	0.8 ± 1.0	2.05 ± 1.3	0.81 ± 1
9956	HGNC:5195	*3OST2*/*HS3ST2*	Heparan Sulfate-Glucosamine 3-*O*-Sulfotransferase 2	0.84 ± 0.35	0.22 ± 0.31	0.24 ± 0.39
9394	HGNC:5201	*6OST1*/*HS6ST1*	Heparan Sulfate 6-*O*-Sulfotransferase 1	66.6 ± 13.9	43.6 ± 49	11.2 ± 11
90161	HGNC:19133	*6OST2*/*HS6ST2*	Heparan Sulfate 6-*O*-Sulfotransferase 2	86.8 ± 19	42.6 ± 43	39.3 ± 77
23213	HGNC:20391	*SULF1*	Sulfatase 1	2.01 ± 2.1	1.2 ± 1.3	0.56 ± 0.88
55959	HGNC:20392	*SULF2*	Sulfatase 2	0.37 ± 0.44	0.37 ± 0.43	0.85 ± 0.81
10855	HGNC:5164	*HPSE*	Heparanase	0.51 ± 0.007	0.39 ± 0.058	0.44 ± 0.35

**Table 2:** Clinical characteristics of patients used in the present study.

Factors	*n* (Total = 42)	*%*
Age		
Median (range)	48.5 ± 12.1	
>48.5	22	54.8
≤48.5	20	45.2
Sex		
Male	19	45.2
Female	23	54.8
Tumour Grade		
II	9	23.8
III	5	11.6
IV	28	66.6
Relapse		
Yes	8	19.0
No	34	81.0
Tumour development		
Primary	27	64.3
Secondary	15	35.7
Death		
Yes	31	73.9
No	11	26.1

**Table 3:** Primer sequences.

Gene	Sequence
*EXT1*	F 5′-AGCACAAGGATTCTCGCTGT-3′
	R 5′-GGAACCAGACAGAAAGTGGC-3′
*EXT2*	F 5′-GTATTTTGCAGAGCATCCCC-3′
	R 5′-GGGCAATGGCTTTAATTGAC-3′
*NDST1*	F 5′-GTATGTCAACCTGGACGCCT-3′
	R 5′-CACTCAGCAGGCTGTTCTCA-3′
*NDST2*	F 5′-TGGTCCAAGGAGAAAACCTG-3′
	R 5′-GTGCAGGCTCAGGAAGAAGT-3′
*GLCE*	F 5′-CACCCCTTCTCTGCTGTCTC-3′
	R 5′-GAATCAAAACTTCAGTTTCTTGTCA-3′
*2OST1/HS2ST1*	F 5′-CCAGATCCAGAAACTGGAGG-3′
	R 5′-TCCATTGTATGTCGCTGCTC-3′
*3OST1/HS3ST1*	F 5′-CAGGAGCCTATTTAGGGCG-3′
	R 5′-AGCAGGGAAGCCTCCTAGTC-3′
*3OST2/HS3ST2*	F 5′-ACCCCACTTCTTTGACAGGA-3′
	R 5′-CAAAGTAGCTGGGCGTCTTC-3′
*6OST1/HS6ST1*	F 5′-CGACTGGACCGAGCTCAC-3′
	R 5′-GGTCTCGTAGCAGGGTGATG-3′
*6OST2/HS6ST2*	F 5′-TCACCAGCTGTGTGCCC-3′
	R 5′-GTGTCGGAGGATGGTGATGT-3′
*SULF1*	F 5′-ATGCAGGTTCTTCAAGGCAG-3′
	R 5′-ATCCTGGTTGAATAATCAATCTCT-3′
*SULF2*	F 5′-CAATAGCACTGGCTACCGGA-3′
	R 5′-TTTTTAAGGAGTCCGACCCA-3′
*Heparanase-1/HPSE*	F 5′-TTCCTGTCCGTCACCATTG-3′
	R 5′-TACGCAGGAGACAAGCCTCT-3′
*GAPDH*	F 5′-AAGGTGAAGGTCGGAGTCAA-3′
	R 5′-AATGAAGGGGTCATTGATGG-3′

## Abbreviations

HS	Heparan Sulfate
HSPG	Heparan Sulfate Proteoglycan
GBM	Glioblastoma Multiforme
ECM	Extracellular Matrix
TME	Tumour Microenvironment
